# Real-Time Correlates of Phonological Quantity Reveal Unity of Tonal and Non-Tonal Languages

**DOI:** 10.1371/journal.pone.0012603

**Published:** 2010-09-08

**Authors:** Juhani Järvikivi, Martti Vainio, Daniel Aalto

**Affiliations:** 1 Max Planck Institute for Psycholinguistics, Nijmegen, The Netherlands; 2 Institute of Behavioral Sciences, University of Helsinki, Helsinki, Finland; 3 Institute of Mathematics, Aalto University, Espoo, Finland; Stanford University, United States of America

## Abstract

Discrete phonological phenomena form our conscious experience of language: continuous changes in pitch appear as distinct tones to the speakers of tone languages, whereas the speakers of quantity languages experience duration categorically. The categorical nature of our linguistic experience is directly reflected in the traditionally clear-cut linguistic classification of languages into tonal or non-tonal. However, some evidence suggests that duration and pitch are fundamentally interconnected and co-vary in signaling word meaning in non-tonal languages as well. We show that pitch information affects real-time language processing in a (non-tonal) quantity language. The results suggest that there is no unidirectional causal link from a genetically-based perceptual sensitivity towards pitch information to the appearance of a tone language. They further suggest that the contrastive categories tone and quantity may be based on simultaneously co-varying properties of the speech signal and the processing system, even though the conscious experience of the speakers may highlight only one discrete variable at a time.

## Introduction

The ability to understand words in natural speech entails rapid conversion of acoustic phonetic information into meaning. The most obvious cues to this end would be features associated with segmental quality and voicing (pig, big, peg, beg) that are exploited by a vast array of languages [1]. In addition, however, a number of languages exploit suprasegmental prosodic devices, such as variation in duration and fundamental frequency (f0), in signaling phonological categories distinguishing word meaning [2], [3]. Importantly, duration and f0 variation have often been seen as mutually exclusive ways to express phonological categories [3], [4]: whereas the latter is used by and associated with so-called tone languages like Mandarin Chinese [5], segmental duration has been taken as the main vehicle to signal lexical contrasts in so-called quantity languages [6], [7 for an overview], of which Finnish is an often cited example [3], [7]–[10]. Therefore, presence or absence of phonological-lexical tone forms one of the fundamental divisions in the (phonological) typology of languages [11], [12].

At the outset, dividing languages into types according to their sound structure seems fairly straightforward: For the speakers of tone languages - such as Mandarin Chinese - the physically continuous changes in pitch appear as distinct tones that determine the lexical meaning of a segmental string (e.g., Chinese *mā* with high level tone “mother” vs. *mă* with falling-rising tone “horse”); the speakers of quantity languages experience sounds as categorically long or short and changes in speech segment duration can crucially differentiate word meaning (Finnish *tuli* “fire” vs. *tuuli* “wind”). Importantly, then, the *forms* by which we consciously experience language seem to be first and foremost based on phonological phenomena like segments, tones or length opposition [13]. Therefore, phonological phenomena such as these arguably form the qualia of our linguistic experience. They appear discrete and categorical, much like colors in visual perception, even though the physical representation underlying the conscious perception is in fact continuous [13], [14]. The division of the continuous physical spectrum into specimens of discrete categories is dictated partly by the system we have learned in the course of our experience. Thus for the speakers of tone languages, f0 variation signals phonological changes that are perceived as differences in lexical meaning. In contrast, it is generally agreed that non-tonal languages use pitch changes (f0 variation) in other functions only; for example, to mark aspects of information structure, like focus, or extra-linguistic phenomena such as emotional content [3], [12]. This would then be the case for the speakers of languages, such as Finnish, that have quantity, but no tonal system (hence, quantity languages): Whereas they would be sensitive to durational information in lexical perception [15], for them pitch changes would be functional only outside the lexical domain. In other words, as is generally assumed, in Finnish pitch would be excluded from signaling formal categories at the lexical level [7], [16].

In this vein, speakers may get tuned to their native phonological categories to the extent that they may have insurmountable problems identifying contrasts that are alien to their native language system [17]. Importantly, research has shown that this is the result of linguistic experience; early on infants are sensitive to a variety of phonetic differences, like segmental (e.g., l-r) or suprasegmental (e.g., stress placement) contrasts [17], [18]. However around six months of age they start losing the ability to discriminate between contrasts that are not part of the phonology of their first language; instead, they start tuning more and more into their native language contrasts [19]–[21]. The end result is that adults, in effect, cannot hear the phonetic differences underlying phonological contrasts that are not native to their mother tongue, such as nuances of lexical tones for speakers of English or lexical stress for speakers of French [17 for an overview]. The extent to which this is the case may depend on the devices present in the speakers' native language. For example, even if stress may usually be realized by a combination of loudness, pitch and duration [4], whether the language in question employs all or only some of these, may depend on its phonological structure: thus, for example, speakers of quantity languages would be expected to avoid duration and speakers of tone languages pitch in this function [4]. Taken together, the evidence suggests that speakers of non-tonal languages should not be able to “hear” lexical tones, or should at least have grave difficulties in doing so. The implication for our purposes would be that speakers of Finnish, considered a prototypical (non-tonal) quantity language, would thus not be expected to be sensitive to these tonal differences.

This qualia-like, categorical nature of our linguistic experience is directly reflected in the traditionally clear-cut linguistic classification of languages into tonal or non-tonal, and, importantly, also underlies the recent hypothesis that the geographical distribution of the alleles of certain genes involved in brain development, namely *MCPH1* and *ASPM*, would be causally related to whether the speakers are biased towards acquiring and processing tonal contrasts and, thus, to the typological distribution of tonal languages [22].

In contrast, we would like to argue that duration and pitch are fundamentally interconnected: more precisely, that, underlyingly, the two apparently mutually exclusive phonological linguistic systems of tone and quantity languages that are associated with the physical parameters of pitch and duration, may be more similar than we have realized at the level of the processing/perceptual mechanisms. We believe that there are a number of observations in the literature that would support this hypothesis: First of all, it is well known that, other things being equal, dynamic and low tones tend to be perceived as longer than high or static ones [23]–[24]. This may be a general psycho-physiologically based propensity characteristic of the human perceptual system [25]–[26], and may perhaps be related to the general tendency in human perception of time to perceive dynamic events in general as taking longer than static events [27]. Interestingly, though, some studies suggest that this association may be stronger for speech than non-speech sounds [28] and maybe especially pronounced for languages with phonological quantity or tone [29]. Secondly, the available evidence suggests that at least in some languages with phonological quantity, the long sounds tend to be coupled with dynamic f0 movement, and, vice versa, in tone languages in general, contour tones tend to be longer in duration than level tones [30]. Moreover, in languages that have both a tonal system and contrastive vowel length, dynamic contour tones are usually restricted to long syllables alone [30], [31]. Thirdly, some evidence suggests that in non-tonal quantity languages such as, Estonian, Finnish, Japanese, and Serbo-Croatian, tonal differences affect speakers' judgments of vowel length [7], [29], [32], [33], in so far as the available evidence can be taken to suggest that the speakers of these languages tend to categorize syllables or words as long more often than short when the target syllable has a falling rather than a level tone. Finally, this intricate relation between duration and f0 movement/pitch - whether fundamentally linked to the basic principles of our perceptual organization or connected to language perception - may also underlie the seemingly sudden changes of phonological systems from a tonal system to one based on quantity, as in the well documented case of Korean [34].

Taken together, the available evidence suggests that at the level of consciously inaccessible perceptual mechanisms, duration- and tone-based sound systems may be tightly related, and that duration and pitch may co-signal semantic distinctions even in languages with no overt system of lexical tones. Underlyingly, then, the two prosodic dimensions may in fact represent two sides of the same coin. In other words, in language perception, these two prosodic principles, duration and tone, could still operate jointly, even though only one of them could be usually available to conscious perception at a time. This predicts that speakers of non-tonal (quantity) languages might be sensitive to tonal differences in perception. Furthermore, provided that the physical-phonetic expression of phonological quantity would be dependent on a systematic coupling of both duration and pitch variation [35], the speakers of quantity languages should be affected by both of these cues when consciously evaluating the “length” of given speech sounds. More crucially, however, if these two systems were intimately linked in production, we would expect pitch information to be used systematically as a cue in rapid automatic real time language perception. In what follows, we will scrutinize these assumptions for Finnish, a prototypical quantity language.

We set out to investigate whether tonal movements affect the perception of quantity in Finnish in offline categorization (Exp. 1) and in online word recognition (Exp. 2) when f0 and duration information were systematically manipulated.

Experiment 1 used a two-alternative forced choice (2AFC) categorization task where participants had to indicate for each auditory stimulus whether they thought the first syllable of the word was “short” or “long”. The materials consisted of five minimal pairs of familiar Finnish words differing with respect to the vowel length of the first syllable only (e.g., *sika* “pig” vs. *siika* “whitefish”). Both members of each pair were spoken by a female speaker and recorded. The first syllable vowel duration of each word was manipulated in five steps (75, 100, 125, 150, 175 ms) and each had either a level or a falling tone. In addition, the second syllable duration was set either to 50 or 100 ms. Altogether this resulted in 200 trials.

Experiment 2 investigated online word recognition using classic cross-modal auditory-visual repetition priming. In cross-modal priming, participants hear a spoken prime word and immediately after the prime offset see a visual target letter string on a computer screen to which they make a lexical decision (word - yes; not word - no). Participants' decision latencies (i.e., reaction times) and response accuracy are recorded. Differences in priming effects measure immediate changes in the activation levels of the target word's mental representation in real time: greater facilitation reflects closer fit between the prime and the target's lexical representation [36]. Importantly, if the presence or absence of pitch movements is decisive for the occurrence and/or degree of facilitation, this indicates that pitch information is used in distinguishing two lexical forms in Finnish, thus constituting a phonologically relevant contrast [37].

The prime-target sequences consisted of thirty minimal pairs of familiar disyllabic nouns differing only with respect to the phonological length of the first syllable vowel. The short spoken member of the pair (*sika* “pig”) was recorded and manipulated: the duration of the first syllable was set to either 110 (short) or 135 (long) milliseconds with either a level (high) or a linear falling (fall) tone. The visually presented targets were words with a “long” first syllable vowel (*siika* “whitefish”). Note that in Finnish word stress is always on the first syllable. Furthermore, it is important to note that the decision to use the short member of each pair to construct the auditory prime stimuli ensured that any segmental and/or microprosodic information left in the stimuli would bias towards *short* rather than *long* interpretation, and, thus, work against rather than in favor of the above hypothesis that a falling tone would *ceteris paribus* facilitate the recognition of these words with long first syllable.

## Results

### Experiment 1

A bivariate logistic regression model [38] was fitted to the data with Type of Source Word (CVV or CV first syllable), First Syllable Duration (75, 100, 125, 150, 175 ms), Second Syllable Duration (50, 100), and Type of Tone (High, Fall) as predictors and the binary response (short, long) as the dependent variable. The model with the best fit to the data is depicted in Tables 1 and 2.

**Table 1:** Summary of results from the logistic regression analyses for Experiment 1 for the model with the best fit.

	Estimate	Std.Error	z value	Pr(>|z|)
(Intercept)	−3.4639	0.2063	−16.787	<2e-16 ***
sourcecvv	1.3455	0.1177	11.428	<2e-16 ***
tonefall	2.2491	0.2033	11.065	<2e-16 ***
dur_s1ms125	2.1756	0.2031	10.712	<2e-16 ***
dur_s1ms150	4.2286	0.2327	18.172	<2e-16 ***
dur_s1ms175	6.4592	0.4042	15.979	<2e-16 ***
dur_s1ms75	−2.8409	0.6046	−4.699	2.62e-06 ***
dur_s2ms50	0.9617	0.1141	8.430	< 2e-16 ***
tonefall∶dur_s1ms125	0.3740	0.2899	1.290	0.197131
tonefall∶dur_s1ms150	0.1193	0.4578	0.261	0.794368
tonefall∶dur_s1ms175	−1.9528	0.5844	−3.341	0.000834 ***
tonefall∶dur_s1ms75	0.3818	0.6409	0.596	0.551394

Notes: Significance codes: 0 ‘***’ 0.001 ‘**’ 0.01 ‘*’ 0.05 ‘.’ 0.1 ‘ ’ 1.

Reference levels for predictors were as follows: tone  =  high, duration of the first syllable (dur_s1)  = 100 ms, duration of the second syllable (dur_s2)  = 100 ms, source word type (source)  =  cv. *df*  = 3799, AIC  = 2182.6.

**Table 2:** Summary of the significance values for the factors source, tone, durations of the first syllable, and duration of the second syllable in Experiment 1 as obtained from the analyses of the deviance Table 1 (function anova(object, test  =  “Chisq”) in R).

	Df	Deviance	Resid.Df	Resid.Dev	P(>|Chi|)
Null			3799	5153.4	
source	1	54.1	3798	5099.3	1.897e-13 ***
tone	1	171.5	3797	4927.7	3.399e-39 ***
dur_s1	4	2681.3	3793	2246.5	0.0000000 ***
dur_s2	1	74.3	3792	2172.2	6.687e-18 ***
tone∶dur_s1	4	13.6	3788	2158.6	8.810e-03 **

Notes: Significance codes: 0 ‘***’ 0.001 ‘**’ 0.01 ‘*’ 0.05 ‘.’ 0.1 ‘ ’ 1.

**Figure 1:**
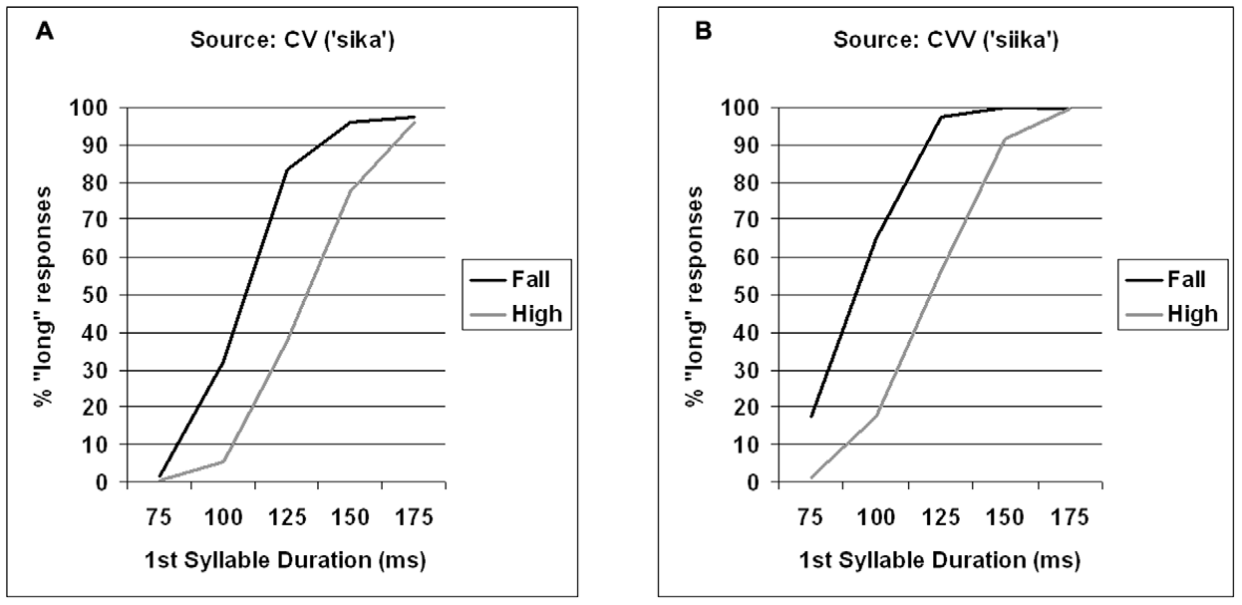
Effects of first syllable duration and Tone in Experiment 1. Effects of first syllable duration (75–175 ms) and type of tone (high, fall) in Experiment1 for source words with short (panel A) and long (panel B) syllables.

**Figure 2:**
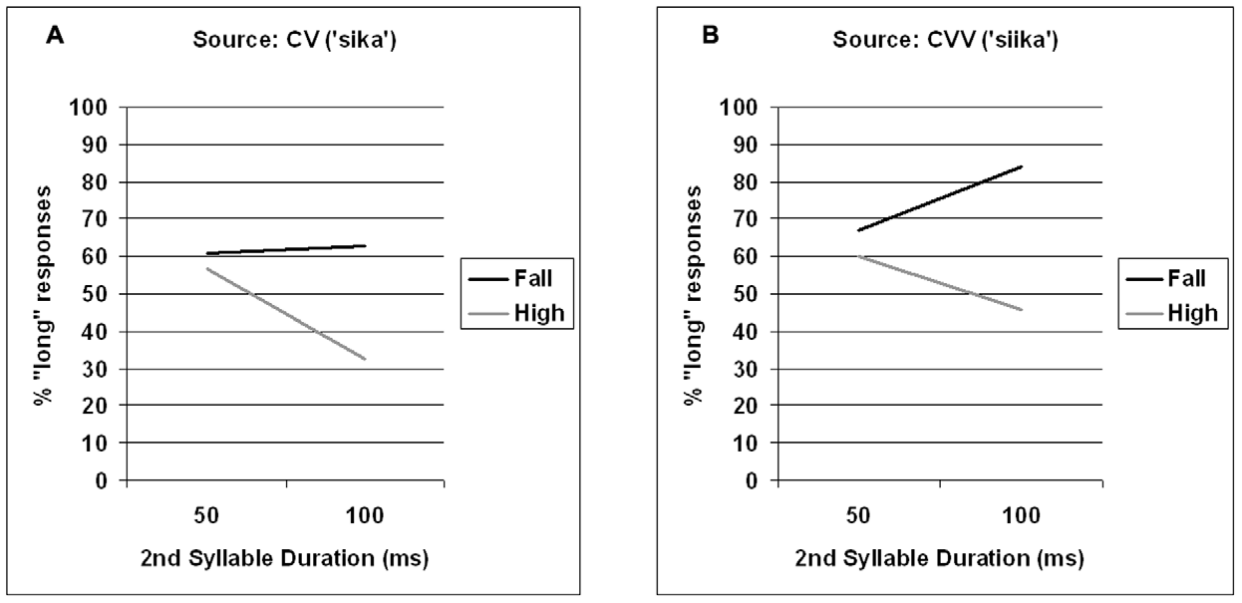
Effects of second syllable duration and Tone in Experiment 1. Effects of second syllable duration (75–175 ms) and type of tone (high, fall) in Experiment1 for source words with short (panel A) and long (panel B) syllables.

As shown in Figure 1, the duration of the first syllable was a highly significant predictor of the distribution of the short-long responses: expectedly, the longer the duration the more ‘long’ choices there were. In addition, whether the second syllable was 50 or 100 ms long affected the participants' choices; namely, as shown in Figure 2, there were fewer ‘long’ responses for the first syllable with the longer of the two durations for the second syllable. This can be expected based on the observation that long first syllables tend to come with shorter vowels on the second syllable than short first syllables [39]. Our results show that listeners pick up on this cue in perception as well. Most notably, however, whether the stimuli had a high level or a falling tone had a robust effect on whether the listeners categorized the first syllable as short or long. More precisely, there were significantly more ‘long’ responses with the falling than the high tone, as shown in Figure 1.

Notably, whether the source word of the stimulus manipulation was the long (CVV) or the short (CV) counterpart did not interact with any of the predictors, showing that the effect of tone was equally strong independent of the source, even though there were overall more ‘long’ categorizations with CVV than CV source words, as shown by the significant main effect (Table 2). However, the type of Tone did interact with the first syllable duration. As shown by Table 1 (see also Figure 1), however, the interaction was mainly due to the type of tone not affecting the perception of the length of the first syllable in the extreme 175 ms category. Moreover, judging by Figure 1 this may have been at least partly due to a ceiling effect. Also, the most extreme short category (75 ms) shows a similar pattern with the short (CV) source words, indicating that pure syllable duration had its clearest effect at both extremes.

To summarize, even though the duration of the first syllable was a significant predictor of the categorization results, it was by far not the only cue to this end: speakers' perception of quantity depended significantly on the duration of the syllable following the first stressed one, and, robustly on whether the first syllable had a level or a falling tone.

### Experiment 2

**Figure 3:**
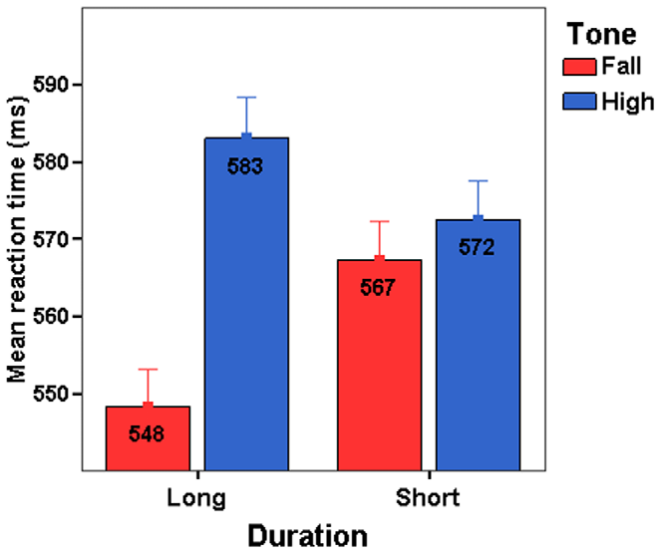
Mean reaction times in Experiment 2. Mean reaction times in milliseconds as a function of tone (high, fall) and duration (short, long) in Experiment 2. Error bars depict 95% CI.

The results from the cross-modal priming experiment are summarized in Table 3 and Figure 3. Before data analyses, erroneous responses as well as latencies that were 2.5 SDs above or below the individual means were removed (altogether 6.7%). One-way analyses of variance for participant means averaged over items (F1) and item means averaged over participants (F2) revealed a significant main effect of priming condition (Table 3) in the response latencies (F1(4,116)  = 6.67, p <.001, F2(4,116)  = 6.44, p <.001). In order to further assess the effects of tone and duration, we inspected the response latency data using 2×2 repeated measures analyses of variance for participants (F1) and items (F2) with Tone (high, fall) and Duration (short, long) as factors (see Table 3). We observed a statistically significant main effect of tone (F1(1, 29)  = 4.79, p <.05, F2(1, 29)  = 7.34, p <.005) indicating more facilitation for the falling than the high tone conditions. There was no significant effect of duration (F1 <1; F2 <1) and no statistically significant interaction (F1  = 1.57, p  = .22; F2  = 3.83, p  = .062). Further one-way analyses of variance revealed no significant differences in the error rates between the five priming conditions ((F1(4,116)  = 1.93, p  = .11; F2(4,116)  = 1.82, p  = .13). We also analyzed the data using linear mixed-effects models with participants and items as a crossed-random factor [38], with Tone and Duration as fixed predictors and subject list and item group as covariants. The results revealed a significant effect of Tone (*β*  = 26.037, *t*  = 2.183, *pMCMC*  = 0.028), but no effect of Duration (*β*  = 17.384, *t*  = 1.456, *pMCMC*  = 0.145), and no significant interaction (*β*  = −22.452, *t*  = −1.335, *pMCMC*  = 0.183).

**Table 3:** Response latencies and error percentages from the priming experiment.

Tone	Duration	Errors (%)	RT (ms)	Priming (ms)
High level	Long	5.2	583	+33
High level	Short	4.6	572	+44
Fall contour	Long	6.9	548	+68
Fall contour	Short	6.3	567	+49
Unrelated	Unrelated	11.5	616	-

Positive sign in the column for Priming indicates facilitation (in ms) compared to the unrelated control condition.

The results are clear: whether the first syllable has a falling or a level (high) tone is a robust online cue to the lexical identity in Finnish. It is important to note that in the present experiment, the recognition of words with long first syllables was significantly facilitated by falling tone despite the fact that the spoken stimuli were constructed on the basis of the short member of the minimal quantity pair. Furthermore, as the results from Experiment 1 indicated, pitch information can be decisive in the absence of sufficient durational cues. As the time constraints of natural speech are bound to affect how duration alone can be realized and detected, we may assume that this situation would not be at all exceptional in real life.

## Discussion

In contrast to the usual assumption that there is a clear-cut conceptual distinction between tone and non-tonal quantity languages, we have put forth the idea that, cognitively, these two phonological systems could perhaps be seen as two variants of using the same underlying mechanisms. In addition to reviewing the available evidence that we thought would point this way, we carried out two experiments investigating whether pitch information would affect perception of length and thus word recognition in a language with a *par excellence* example of a quantity-based lexical-phonological system. The answer based on the two experiments was a clear affirmative.

Experiment 1 showed that, in addition to duration, participants' categorization of syllables as long or short was robustly dictated by whether the relevant (first) syllable had a level or a falling tone corroborating similar observations in previous studies [7]. Furthermore, there is evidence suggesting that our result would apply (at least) to quantity languages more generally [29], [32], [33]. Interestingly though, the results also showed that listeners picked up on the second syllable duration as a cue to the quantity difference. This further indicates that systematic tendencies observed in production data, e.g., the variation of the second syllable duration *vis-à-vis* the first syllable quantity [39] and the related tonal differences [35], affect speakers' speech comprehension irrespective of whether they are aware of these phenomena or not. Even though duration is a salient feature for a Finnish speaker, as shown also here, its prominence as *the* experiential correlate of quantity is no doubt enforced by the fact that quantity is systematically marked in Finnish orthography by doubling the corresponding grapheme (e.g., tuli “fire” vs. tuuli “wind”; tuli “fire” vs. tulli “customs”); and, further enhanced by the fact that Finnish has a shallow orthography, i.e., in popular conception a word is thought to be pronounced exactly as written. More importantly, however, the result from Experiment 1 extended to Experiment 2 where we showed for the first time that the effect of tonality is not restricted to simple offline categorization, but is systematically and automatically used by speakers in rapid online word recognition to identify phonological quantity.

First, our results showed that pitch information is an important co-index of the quantity opposition in Finnish. The fact that it can modulate online word recognition suggests that this information may be coded in the Finnish mental lexicon. Further experimentation is needed, however, to determine when in the recognition process pitch information is used. Consequently, however, our results imply that in terms of the production and perception *mechanisms*, pitch in Finnish is probably in all respects like pitch in any prototypical tone language, e.g., Mandarin Chinese. In other words, even though in these languages the conscious experience of the speakers may highlight only one discrete variable, length or tone, at a time, the results indicate that from the point of view of the processing system, the contrastive categories of tone and quantity may be based on the same simultaneously co-varying properties of the speech signal.

Traditionally, the distinction between phonetics and phonology has been defined by asserting that the former is associated with the distribution of physical patterns in a speech signal, for example, duration or pitch, whereas the latter is usually concerned with what is taken as the psychologically relevant abstract sound category represented in the speakers' mind, e.g., quantity or tone [40]. In other words, whereas phonetic phenomena present themselves as inherently continuous, phonological patterns are often seen as discrete and categorical [41]. Consequently, traditional phonological generalizations have usually highlighted the experientially most salient phonetic correlates; for example quantity-*qua-*duration, as in the case of Finnish. The consequence has been that those features that are experientially/consciously accessible to native speakers have often been thought of as the most important - if not the only - features underlying the actual realization and recognition of phonological categories [42], [43]. Therefore, the most obvious corresponding physical parameters, e.g., duration or pitch, have sometimes been assumed to be the necessary and sufficient features underlying the phonological distinction, as for quantity and duration in the case of Finnish [3], [10], [39]. However, recent research already indicates that many, seemingly robust, phonological phenomena are modulated by an interplay of a variety of phonetic cues in perception [44]. The present results - in so far as they suggest that tone information is coded in Finnish mental lexicon - further call for concurrent use of different sources of data, including experimental, if we are interested in a truly cognitively motivated linguistic theory of sound structure e.g., [45].

Finally, in a recent study Dediu and Ladd [22] observe a significant correlation between the distribution of allelic forms of the ASPM and microcephalin (MCPH) genes in the human population and the distribution of tone languages within the respective subpopulations. More specifically, they note that the population frequencies of the more recent derived haplotypes, ASPM-D and MCPH-D, correlate negatively with the use of linguistic tone. Tone, in turn, would then presumably be associated more with the ancestral forms of these two genes. Even though they take pains to stress that it would be a question of these genes inducing a very small, non-deterministic, probabilistic cognitive bias that would facilitate the ability to acquire as well as process pitch information, they do interpret this result in terms of a causal chain from the presence of the prospective allelic variant via the caused bias in learning and processing of pitch information to the distribution of tone languages (to put it in a vastly simplified manner). Even though the work has been taken as generating hypotheses more than giving specific answers [46], it does imply the hypothesis that an individual speaker's phenotype will causally affect their ability to acquire and process - hear - tonal distinctions [47]. More importantly, however, the argument takes it that sufficiently large numbers of certain allelic forms in a human population might lead to the birth of a tone language. However, our results suggest that there is no unidirectional link from the actual perceptual information - pitch or duration - used in processing to mark phonological distinctions to the experientially based language type classification. In other words, the use of pitch to mark and process lexical-phonological distinctions in a language does not entail that the language be categorized as a tone language. This is not, of course, direct counter evidence, if such could exist, to the above hypothesis simply for logical reasons. Also, it is not straightforward to come up with a direct way to test whether people with genotypes associated with tone languages and non-tone languages would be equally sensitive to tone information without the confounding influence of language background entering the picture; thus, direct conclusive evidence is difficult to find. Rather, our result raises the important question of the relationship between biologically and psychologically based properties of speaker populations and typological linguistic generalizations.

Putting aside the fact that any causal inference from physiological factors to (largely) normative, discrete, categories like language types is problematic *per se*, what our results show is that the phonological quantity system in Finnish is not based on (the perception of) duration alone, but that both pitch and durational information in fact codetermine the length opposition. In other words, the Finnish quantity system cannot be based on an *a priori* heightened sensitivity to acquire and process durational information, but requires sensitivity to pitch information as well. It is of course possible, however, that even though Finnish speakers are clearly sensitive to pitch, they could not still be *less* sensitive to pitch than e.g., speakers of prototypical tone languages. Thus, pitch sensitivity could be a continuous trait that affects cross-population variance with respect to the sensitivity to tone information; some speakers may carry alleles that make them relatively more sensitive to tone information; or, vice versa, Finnish speakers might carry alleles that make them less sensitive to tone information than speakers of tone languages.

Furthermore, even though we have argued that tone and (non-tonal) quantity languages might represent two facets of a common perceptual system, it is an open question to which extent Finnish speakers' sensitivity to pitch follows from a learned association between pitch and duration in this language. Even if much evidence suggests that pitch and duration are intimately connected in perception more generally, this does not mean that the two possibilities are mutually exclusive; nor does it mean that the more deep rooted connection and any learned association could not contribute to the balance of pitch and duration to different degrees and interact with other factors, like language experience or genotype. Therefore, we would like to argue that rather than a discrete categorical classification of languages into tone languages and non-tone languages, a more fine-grained account is needed [48] that takes into account the extent to which (in this case) pitch information is actually used to distinguish phonological categories in processing. This would not only sharpen our criteria of tone languages, but would also provide a more realistic, more refined, explanandum for studies of linguistic evolution.

To conclude, we showed that speakers of a *par excellence* prototypical non-tonal quantity language use pitch information in speech perception as a cue to the length opposition. Although duration and pitch are exploited in parallel, pitch can be the primary cue in activating the right lexical candidate. We take our results to suggest that tone and quantity languages may have a common perceptual basis. In other words, we interpret our results as showing that these contrastive categories, tone and quantity, are marked by the same simultaneously co-varying properties of the speech signal and the processing system even though the conscious experience of the speakers may highlight only one discrete variable at a time. This further indicates that the process of language change may be masked from the experience of the speaker population until it reaches a state in which one of the underlying perceptual variables has become the primary cue for a given contrast. With regard to tonogenesis - at least in some cases - it may be that tone in the phonetic sense has been present all along and only surfaces phonologically when other linguistic factors force the change. Importantly, our results suggest that there is no unidirectional link from perceptual sensitivity to pitch information to the emergence of a tone language. Whether or not a causal link exists between the genetic endowment of a given subset of the human population and certain linguistic properties, any cognitively motivated approach to a phonological contrast like quantity or tone should account for the interplay of voice fundamental frequency and segmental duration in perception and acquisition and the perceptual commonality of tone and quantity languages.

## Materials and Methods

### Ethics statement

The experiments of the present study were non-evasive and were carried out in accordance with Finnish law and adhered to the guidelines of the American Psychological Society and the ethical policies of the University of Helsinki. The present type of (non-medical) research is exempt under Finnish legislation for ethical review and approval. Participants gave written informed consent to their participation.

### Quantity in Finnish

Finnish is standardly thought of as a prototypical (non-tonal) quantity language, i.e., a language in which durations (of segments) are primarily used to distinguish word meaning [3]–[10]. Accordingly, Finnish exhibits two degrees of length for most sounds of the language in most positions: thus, virtually any phoneme - vowels in all positions and intervocalic consonants - can be realized in the short and the long form. Examples abound: tule - tuule - (ei) tulle - (ei) tuulle - tulee - tuulee - tullee - tuullee. Moreover, it is generally accepted that Finnish has neither tone nor stress that would be lexically distinctive [3]. Word stress in Finnish is always on the first syllable. According to the standard view, the quantity opposition can be explained solely by the durations of the different segments and their mutual relationships. In other words, quantity in Finnish - as is often the case in general - is equated with physical duration [see 7 and the references therein]. In this view, there are either no tonal differences between the contrastive quantity categories, or, at least any such differences would be non-systematic and accidental [39]. A recent, more refined, argument to this effect [16] takes it that underlyingly all Finnish word types are systematically uniform with respect to their tonal form. This single tonal pattern is then timed according to the number of morae in the syllable: there is a rise in pitch in the first mora of a word, whereas in the second mora, there is a fall.

Interestingly, however, a number of researchers have noted that the quantity difference in Finnish may be coupled with a difference in fundamental frequency contour [7], [35]. In fact, already in the fifties, Malmberg [49] noted that Finnish long vowels tend to have a falling f0 pattern in contrast to the high level f0 pattern found on the short vowels. More recently, a production study by Vainio et al. [35] lent further credibility for this observation: based on Finnish CV-CVV and CV-CVC word pairs, such as/pu.ro/-/puu.ro/(“stream”- “porridge”) an/ka.ma/-/kam.pa/(“stuff” - “comb”). The study showed that the quantity distinction correlated with a falling tone in long as opposed to a high level tone in short syllable nuclei. In addition to production data, some evidence suggests that speakers may use this information in the perception of length in quantity languages such as Serbo-Croatian, Estonian, Finnish, and Japanese [7], [29], [32], [33]. This suggests that pitch affects offline categorization for these languages, even though the evidence is less than conclusive. Whether this cue affects online word recognition - as one might assume if it is systematically used in production - has not been studied at all. Yet this would be crucial for our understanding of the relation between tonal and durational cues in quantity and tone languages.

### Experiment 1

#### Participants

Nineteen students from the University of Helsinki participated in the experiment. All reported normal hearing.

#### Materials

**Figure 4:**
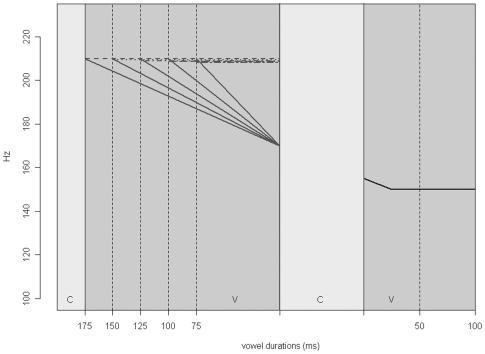
Schematic representation of the construction of materials in Experiment 1.

The materials consisted of five pairs of words differing in the quantity (length) of the first syllable vowels; short (CV) or long (CVV). Orthographically the pairs were as follows: *sika - siika* (“pig” - “perch”), *kisu - kiisu* (“kitten” - “ore”), *Mika - Miika* (Finnish male forenames), *kato - kaato* (“loss” - “felling”), and *pika - piika* (“instant” - “maiden”). The ten words were embedded in a carrier sentence *sano sana X tasaisesti* ‘say the word X evenly’ and spoken by a female speaker. The utterances were recorded in a sound treated room at the Department of Speech Sciences of the University of Helsinki using a high quality AKG 4000B condenser microphone and a high quality Digidesing Digi002 analogue to digital converter. The word segments were isolated from the carrier sentences for further processing which was done using the PSOLA algorithm in the program Praat [50]. The syllable nuclei of the isolated target words were labeled and both f0 and duration were manipulated. The duration of each first syllable nucleus was set to five different values, with a 25 millisecond difference between the values (75, 100, 125, 150, and 175 ms). The actual durations of the vowels were, however, somewhat longer (15–20 ms) due to the fact that the manipulations did not take place throughout the whole vowel. The duration of the second syllable vowel was set to 50 and 100 ms (again with somewhat longer actual durations remaining in the stimuli). The tone of the first syllable vocalic nucleus was set to two different levels: a straight linear (high) tone throughout the vowel or a linear fall. Figure 4 shows a schematic representation of the materials. The f0 of the second syllable was left intact. All in all, this resulted in 10×5×2×2 = 200 stimuli. The manipulation still left the second syllable onset consonant intact. The mean durations of the second syllable onset consonants were 146 ms for the CV words and 120 ms for the CVV words.

#### Procedure

The resulting 200 stimuli were collected together into a long sound file in a randomized order. A two-second silence was inserted between each stimulus (resulting in approximately 2.2 s interval between the trials). After every ten stimulus trials, a short tone was played in order for the participants to be able to check whether they were in synchrony with the test. The participants were given an answer sheet with numbered lines containing the stimulus number and two boxes. They were instructed to mark on the sheet whether they perceived the vowel of the first syllable of the stimulus word to be either “short” or “long” by ticking the appropriate box. A practice block consisting of ten trials preceded the experimental trials.

### Experiment 2

#### Participants

Thirty students from the University of Helsinki participated in the experiment. All reported normal hearing.

#### Materials

Thirty familiar noun-noun minimal pairs differing only in whether the vowel in the first syllable was short or long (*sika* “pig” vs. *siika* “whitefish”) were selected. The auditory primes were constructed using the short member of each pair, e.g., *sika*. This was done in order to ensure that any segmental and/or microprosodic information left in the stimuli after the manipulation would bias towards “short” rather than “long” interpretation, and, would thus make it less rather than more likely that the recognition of the “long” counterpart, e.g., *siika*, would be facilitated due to this information. The prime words were spoken by a female native speaker of Finnish and recorded in a sound treated chamber at the Department of Speech Sciences of the University of Helsinki using a high quality condenser microphone (AKG 4000B) and a high quality and analogue to digital converter (Digidesing Digi002). To produce the desired stimuli the f0 and duration of the words were manipulated as follows: First, all spoken words were segmented similarly to ensure that their onsets and offsets would be as similar as possible. The tonal and durational manipulation was done using pitch synchronous overlap add (PSOLA) method as implemented in the *Praat* phonetic analysis software.

**Figure 5:**
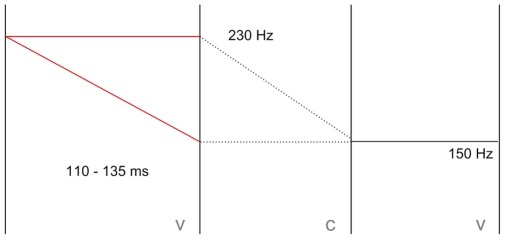
Schematic representation of the construction of materials in Experiment 2.

**Figure 6:**
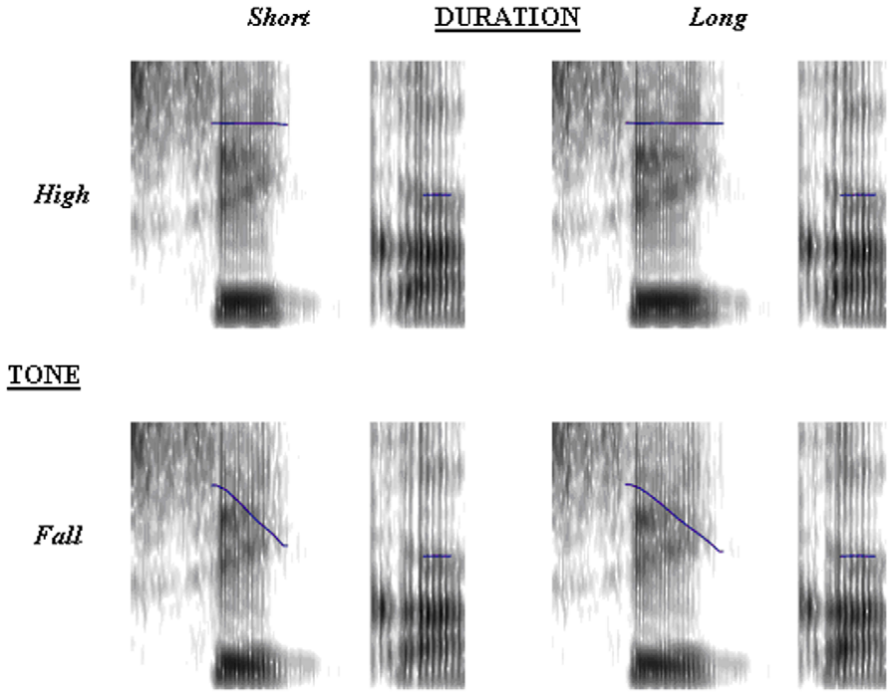
Example of actual materials from Experiment 2. Example of actual materials from Experiment 2 showing spectrograms and f0 contours for tone (high, fall) and duration (short, long) in the critical conditions. Corresponding audio files Audio S1-S4 can be found in the supporting materials.

Four versions of each word token were produced as depicted schematically in Figure 5. The duration of the first vowel was set at either 110 or 135 milliseconds. This range was determined based on the results from Experiment 1, where participants were asked to classify the first syllables of similarly manipulated words into short or long. The duration of the second vowel was left intact and it varied from ca. 75 to 110 ms. Note that, as with the segmental and microprosodic residue left in the first syllable, the information in the second syllable would be more likely to bias perception towards “short” rather than “long” interpretation. The pitch trajectory of the first syllable was either set at 230 Hz or a linear fall from 230 Hz to 150 Hz was inserted. This closely resembles the situation in natural speech. The f0 range (230 to 150 Hz) was determined from the recordings themselves. This resulted in the four critical priming conditions; Figure 6 shows the spectrograms and f0 contours for the four actual experimental stimuli derived from the CVCV word *sika* “pig”. The corresponding sound files Audio S1-S4 can be found in the supporting materials. In addition to the four experimental conditions, a neutral control condition was added where the prime was neither phonologically nor semantically related to the target.

For the cross-modal priming experiment, the critical prime-target pairs were counterbalanced over 5 lists and presented randomized for each participant. Additional 60 prime-target filler trials were added. In order to balance the possibility of “yes” and “no” decisions, 90 trials with pseudoword targets were added. The pseudowords were phonotactically legal pronounceable letter strings created by changing 1–3 letters from an existing Finnish word but were not existing words in Finnish.

#### Procedure

The stimulus presentation and data collection was done using E-Prime® 1.0 stimulus presentation software and the E-Prime Serial Response Box, together providing timing at millisecond accuracy. At the beginning of each trial, a fixation cross was shown for 500 ms followed by the spoken prime through high quality headphones (Sennheiser HD 250). Immediately at the offset of the auditory prime the target string appeared at the center of the screen in black 24 point Chicago letters on light gray background and stayed for 1000 ms or until the participant responded. The participants were instructed to decide whether the letter string appearing on the computer screen was a Finnish word or not by pressing the appropriate “yes” or “no” key on the response box. Response latencies and accuracy were recorded for further analyses.

## Supporting Information

Audio S1 Example stimulus used in Experiment 2, corresponding to Figure 6, condition High-Short.
(0.01 MB MP3)

Audio S2 Example stimulus used in Experiment 2, corresponding to Figure 6, condition High-Long.
(0.01 MB MP3)

Audio S3 Example stimulus used in Experiment 2, corresponding to Figure 6, condition Fall-Short.
(0.01 MB MP3)

Audio S4 Example stimulus used in Experiment 2, corresponding to Figure 6, condition Fall-Long.
(0.01 MB MP3)
